# Role of ROX1, SKN7, and YAP6 Stress Transcription Factors in the Production of Secondary Metabolites in *Xanthophyllomyces dendrorhous*

**DOI:** 10.3390/ijms23169282

**Published:** 2022-08-18

**Authors:** Pilar Martínez-Moya, Sebastián Campusano, Dionisia Sepúlveda, Alberto Paradela, Jennifer Alcaíno, Marcelo Baeza, Víctor Cifuentes

**Affiliations:** 1 Departamento de Formación General, Facultad de Educación y Ciencias Sociales, Universidad Andrés Bello, Santiago 7591538, Chile; 2 Departamento de Ciencias Ecológicas, Facultad de Ciencias, Universidad de Chile, Santiago 7800003, Chile; 3 Laboratorio de Proteómica, Centro Nacional de Biotecnología, C.S.I.C., 28049 Madrid, Spain

**Keywords:** iTRAQ, RNA-seq, ROX1, SKN7, YAP6, glucose, maltose, catabolic repression

## Abstract

*Xanthophyllomyces dendrorhous* is a natural source of astaxanthin and mycosporines. This yeast has been isolated from high and cold mountainous regions around the world, and the production of these secondary metabolites may be a survival strategy against the stress conditions present in its environment. Biosynthesis of astaxanthin is regulated by catabolic repression through the interaction between MIG1 and corepressor CYC8–TUP1. To evaluate the role of the stress-associated transcription factors SKN7, ROX1, and YAP6, we employed an omic and phenotypic approach. Null mutants were constructed and grown in two fermentable carbon sources. The yeast proteome and transcriptome were quantified by iTRAQ and RNA-seq, respectively. The total carotenoid, sterol, and mycosporine contents were determined and compared to the wild-type strain. Each mutant strain showed significant metabolic changes compared to the wild type that were correlated to its phenotype. In a metabolic context, the principal pathways affected were glycolysis/gluconeogenesis, the pentose phosphate (PP) pathway, and the citrate (TCA) cycle. Additionally, fatty acid synthesis was affected. The absence of ROX1 generated a significant decline in carotenoid production. In contrast, a rise in mycosporine and sterol synthesis was shown in the absence of the transcription factors SKN7 and YAP6, respectively.

## 1. Introduction

Over time, the organisms had to adapt their antioxidant systems to protect themselves from environmental conditions. The yeast *Xanthophyllomyces dendrorhous* has been forced to develop several responses to alleviate extreme growth conditions characterized by low temperature, osmotic stress, and ultraviolet radiation, among others. These evolutionary responses include the accumulation of the carotenoid astaxanthin for photooxidative protection [1]. The biosynthetic pathway of this compound includes specific and nonspecific carotenogenic enzymes [2], since the final step to produce astaxanthin from betacarotene is performed by a cytochrome P450 monooxygenase [3], whose activity depends on a cytochrome P450 reductase that is not a carotenogenic enzyme [4]. Interestingly, this yeast has weak catalase activity; it only possesses Mn-superoxide dismutases (MnSODs) and lacks other antioxidant enzymes. These low antioxidant responses appear to be compensated by astaxanthin production [5,6].

This yeast is able to perform a respiratory and fermentative metabolism and to grow in several carbon sources [7,8,9,10]. This metabolic plasticity and its relation with astaxanthin production was studied previously, where significant differences were observed in the protein and carotenoid profiles depending on the harvest time and carbon source employed [11,12]. Currently, the carotenoid synthesis pathway in *X. dendrorhous* is known, as well as the main factors that regulate it, such as oxygen level, temperature, and carbon source [10]. For instance, catabolic repression regulates pigment production; thus, in the presence of glucose, the production of astaxanthin is inhibited [13,14,15].

It is well known that in the presence of glucose, the model yeast *Saccharomyces cerevisiae* prefers fermentative metabolism even in the presence of oxygen. As a consequence, respiration, gluconeogenesis, and the use of alternative carbon sources are reprimed [16]. This effect is transmitted to the cell through specific signaling pathways. The transcription factor MIG1, a Cys2His2 zinc-finger protein, binds to the promoters of several genes and represses their transcription in the presence of glucose [17] by recruiting the general corepressor complex CYC8–TUP1 [18].

The regulatory role of the corepressor systems CYC8–TUP1 and MIG1 in carotenogenesis has been identified in *X. dendrorhous* [14,19]. In a recent work, the effects of deletion of genes *MIG1*, *CYC8*, and *TUP1* were evaluated through a proteomic and transcriptomic approach. It was shown that the deletion of each transcriptional regulator caused characteristic effects in metabolism, suggesting a more complex gene regulation network upon catabolic repression [20]. We postulated that the CYC8–TUP1 complex performs a central role in the interaction with other transcription factors or regulatory genes in *X. dendrorhous* to repress or activate metabolic pathways in response to environmental conditions, which may modulate astaxanthin production.

In fungi, signaling pathways related to several processes, including fungicide sensitivity, cell wall biosynthesis, cell cycle, sporulation, and oxidative stress adaptation, present niche-specific dynamics [21]. In this context, transcription factors play a key role in modulating the stress-specific response and metabolism when faced with unfavorable environmental conditions. However, understanding the metabolic regulation network through those factors is a complex task, especially for *X. dendrorhous*, where knowledge is limited.

Stress-related transcription factors, such as ROX1, YAP6, and SKN7, have been identified in *S. cerevisiae* [22] and interact with the corepressor complex CYC8–TUP1 [22]. Previous studies have shown that ROX1 recruits CYC8 and TUP1 to hypoxic genes under aerobic conditions [23,24]. YAP6 is closely related to AP-1 factors that belong to the family of basic leucine zipper (bZIP) proteins; it is conserved in all eukaryotes [25,26,27] and activates and represses genes in response to salt-promoted osmotic stress [28]. Nevertheless, it has been found that YAP6 is induced by several forms of environmental stress, which suggests a universal role in the stress response [27]. Similar to YAP6, SKN7 is related to osmoregulation, and it is also necessary for the induction of heat-shock genes in response to oxidative stress in *S. cerevisiae* with both activating and repressing functions at the transcriptional level [28,29].

Therefore, in the present study, we evaluated the effect of mutant strains on the stress-related transcription factors ROX1, SKN7, and YAP6 in *X. dendrorhous* in a metabolic context. For these, we utilized a transcriptomic (RNA-seq) and proteomic (isobaric tags for relative and absolute quantification, iTRAQ) approach during the exponential growth phase with two fermentable carbon sources, and its relation to stress-related secondary metabolites was analyzed. The results show that these genes are involved in the regulation of the biosynthesis of astaxanthin and mycosporines in *X. dendrorhous*, which reinforced the hypothesis that the CYC8–TUP1 complex mediates, at least in part, the stress response of this yeast.

## 2. Results

### 2.1. Transcription Factor Characterization of X. dendrorhous

**Figure 1:**
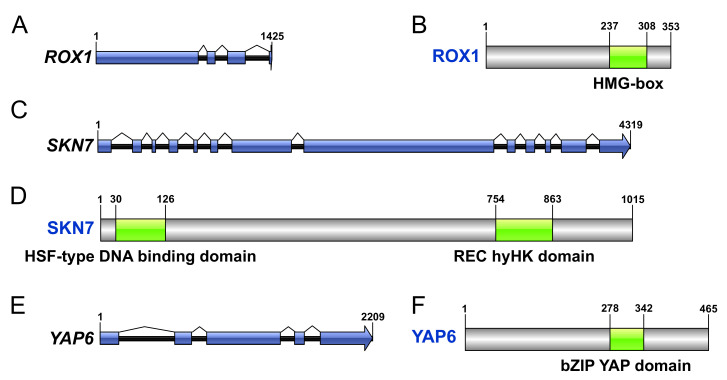
Bioinformatic characterization of *ROX1*, *SKN7*, and *YAP6* in *X. dendrorhous*. Graphical representations of genes *ROX1* (**A**), *SKN7* (**C**), and *YAP6* (**E**) identified in *X. dendrorhous* are shown, where blue linked boxes indicate its coding region as exons. The gene products ROX1 (**B**), SKN7 (**D**), and YAP6 (**F**) are represented with gray boxes, corresponding to their translated sequences, where green regions show significant conserved domains (E-value < 0.01). Each conserved domain and its coordinates are indicated.

The *ROX1*, *SKN7*, and *YAP6* genes were identified in the transcriptome of *X. dendrorhous* based on their similarity to their homologous genes in *S. cerevisiae*. Intron-exon structures were determined, and conserved domains were evaluated. *ROX1* is a gene 1425 bp in length, and its coding region shows four exons (Figure 1A) and encodes a 353 amino acid-length protein with a conserved HMG-box superfamily domain (Figure 1B). Moreover, *SKN7* is a gene that is 4319 bp in length, its coding region shows 13 exons (Figure 1C), and it encodes a 1015 amino acid-length protein. This protein shows a conserved HSF-type DNA binding domain, and a conserved phosphoacceptor receiver (REC) domain (Figure 1D). Finally, *YAP6* is a gene that is 2209 bp in length; its coding region shows five exons (Figure 1E), and it encodes a 465 amino acid-length protein that shows a conserved basic leucine zipper (bZIP) domain of yeast activator protein (YAP) (Figure 1F).

**Figure 2:**
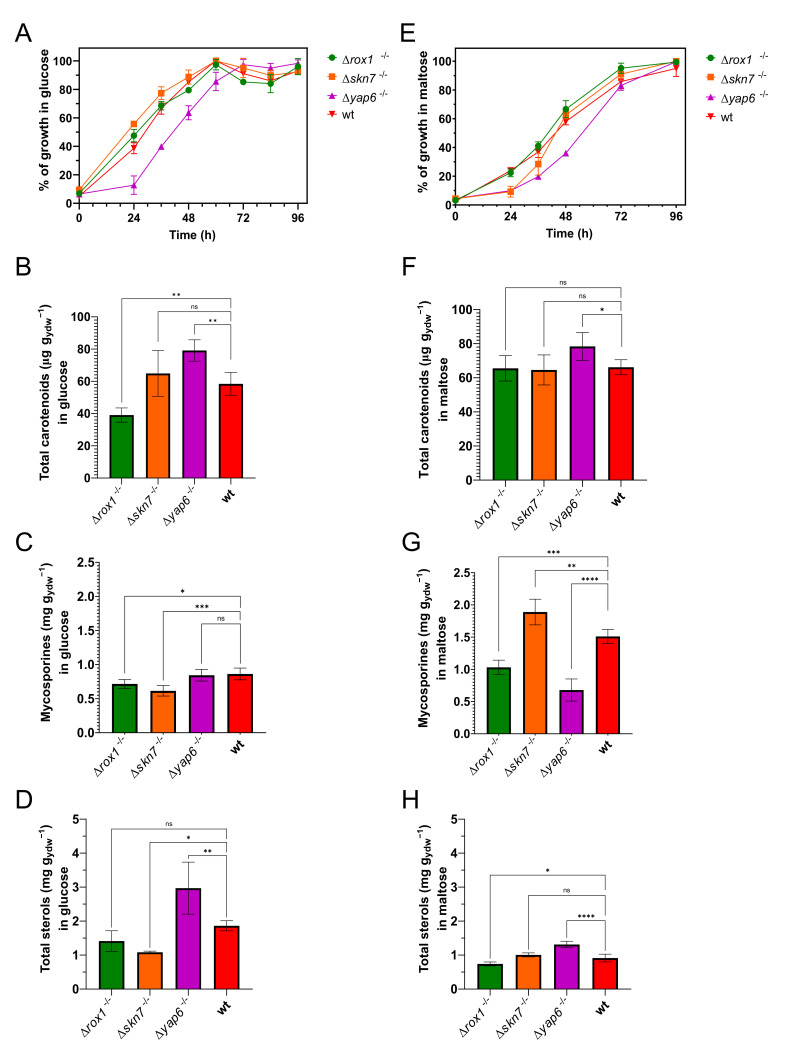
Phenotypic characterization of Δ*rox1*^−/−^, Δ*skn7*^−/−^, and Δ*yap6*^−/−^ mutant strains in *X. dendrorhous*. Growth profile of wild-type and mutant strains in minimal medium YNB glucose 2% (glucose condition, (**A**)) and in minimal medium YNB maltose 2% (maltose condition, (**E**)). It is shown as the percent of maximal growth observed in each strain. Bar plots represent the carotenoid production in glucose (**B**) and maltose (**F**), the mycosporine production in glucose (**C**) and maltose (**G**), and the total sterol content in glucose (**D**) and maltose (**H**) for each strain used in this work. One-way ANOVA followed by Fisher’s LSD test was used for statistical comparison between the wild-type and each mutant strain. Statistical significance is represented with asterisks over each line (* *p* ≤ 0.05, ** *p* ≤ 0.01, *** *p* ≤ 0.001, and **** *p* ≤ 0.0001, ns = not significant).

Deletion mutants for these genes in *X. dendrorhous* were obtained, and phenotypic analyses were carried out, emphasizing their growth profile and secondary metabolite production. Two conditions were studied, in which the yeasts were grown with glucose or maltose as the sole carbon source. Under glucose conditions, the Δ*yap6*^−/−^ strain showed a longer lag phase than the wild type and the other mutant strains studied (Figure 2A). The Δ*rox1*^−/−^ strain showed significantly decreased carotenoid production compared to the wild-type strain, while the Δ*yap6*^−/−^ strain showed an increase in carotenoid content (Figure 2B). Strains Δ*rox1*^−/−^ and Δ*skn7*^−/−^ showed decreased mycosporine production compared to the wild-type strain (Figure 2C). In addition, the Δ*skn7*^−/−^ and Δ*yap6*^−/−^ strains showed decreased and increased total sterol accumulation, respectively (Figure 2D). In contrast, under maltose conditions, the Δ*yap6*^−/−^ and Δ*skn7*^−/−^ strains showed a longer lag phase than the wild-type strain (Figure 2E). Nonsignificant differences were observed in carotenoid production between Δ*rox1*^−/−^, Δ*skn7*^−/−^, and wild-type strain, meanwhile it was increased in Δ*yap6*^−/−^ strain under this condition (Figure 2F). The Δ*skn7*^−/−^ strain showed an increase in mycosporine production, while Δ*rox1*^−/−^ and Δ*yap6*^−/−^ decreased its mycosporine content compared to the wild-type strain (Figure 2G). Finally, under maltose conditions, the Δ*yap6*^−/−^ and Δ*rox1*^−/−^ strains showed an increase and a decrease in total sterol accumulation, respectively (Figure 2H).

### 2.2. Transcriptomic Analysis of Δrox1^−/−^, Δskn7^−/−^, and Δyap6^−/−^ Strains

**Figure 3:**
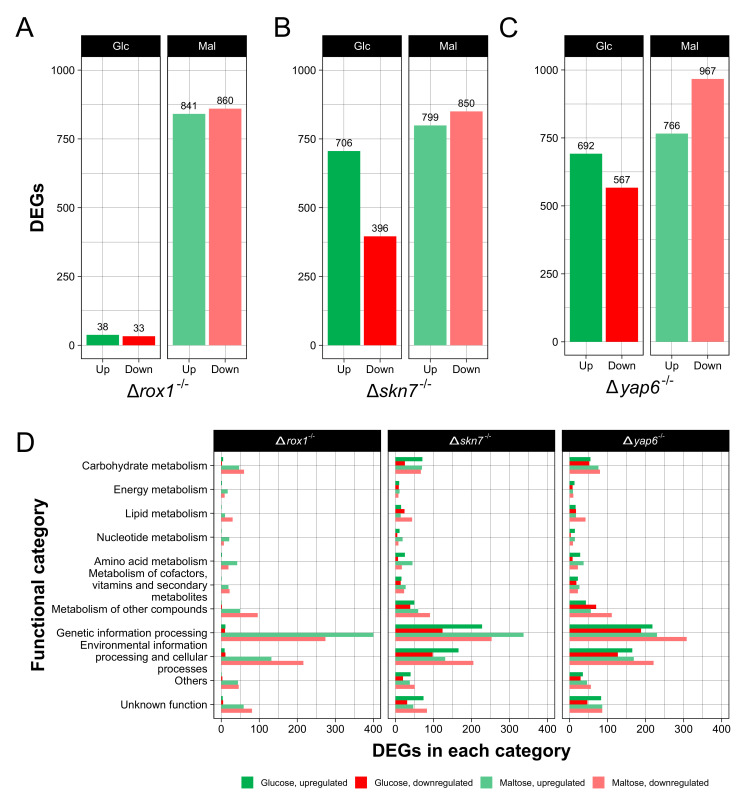
RNA-seq analysis of Δ*rox1*^−/−^, Δ*skn7*^−/−^, and Δ*yap6*^−/−^ strains in *X. dendrorhous*. Mutant strains grown in glucose or maltose were compared with the wild-type strain grown in glucose or maltose, respectively. Differentially expressed genes (DEGs) were determined (absolute log2-fold-change ratio > 1, FDR *p* value ≤ 0.05). The bar plots show the total DEGs identified for the Δ*rox1*^−/−^ (**A**), Δ*skn7*^−/−^ (**B**), and Δ*yap6*^−/−^ (**C**) strains in each condition. The horizontal bar plot shows the total DEGs in each functional category (**D**). Upregulated DEGs (green for glucose, light green for maltose) indicate overexpression in the mutant strain, while downregulated DEGs (red for glucose, light red for maltose) indicate downregulation in the mutant strain.

To evaluate genome-wide regulation by these transcription factors, RNA-seq analyses were carried out comparing wild-type and each mutant strain under both conditions studied (glucose or maltose as carbon source), and differentially expressed genes (DEGs) were identified. A fold change ratio criterion and an FDR *p* value ≤ 0.05 under each condition were employed for data analyses. In the Δ*rox1*^−/−^ strain, 38 and 33 DEGs were found to be upregulated and downregulated under glucose conditions, respectively, and 841 and 860 were found to be upregulated and downregulated under maltose conditions, respectively (Figure 3A). On the other hand, in the Δ*skn7*^−/−^ strain, 706 and 396 DEGs were found to be upregulated and downregulated under glucose conditions, respectively, and 799 and 850 were found to be upregulated and downregulated under maltose conditions, respectively (Figure 3B). Finally, in the Δ*yap6*^−/−^ strain, 692 and 567 DEGs were found to be upregulated and downregulated under glucose conditions, respectively, and 766 and 967 were found to be upregulated and downregulated under maltose conditions, respectively (Figure 3C).

Functional classification of DEGs was carried out based on the KEGG Orthology (KO) database, as previously described [20]. The most represented functional categories between DEGs were “Genetic information processing” and “Environmental information processing and cellular processes” for all mutants in both conditions (Figure 3D). Interestingly, DEGs in “Genetic information processing” were mainly upregulated in all mutant strains in both conditions, except in Δ*yap6*^−/−^ in maltose, where DEGs in this category were mainly downregulated. Other categories, such as “Carbohydrate metabolism” and “Metabolism of other compounds,” were also highly represented ubiquitously between all strains and conditions. “Amino acid metabolism” was mainly upregulated in all mutant strains in both carbon sources. In addition, “Lipid metabolism” was found to be mainly downregulated in the Δ*rox1*^−/−^ and Δ*yap6*^−/−^ strains under maltose conditions, and in the Δ*skn7*^−/−^ strain under both carbon sources. Notably, several DEGs were classified on “Unknown function” category because it was unable to assign a k-number to them.

### 2.3. Influence of ROX1, SKN7, and YAP6 Deletion on X. dendrorhous Proteome

**Figure 4:**
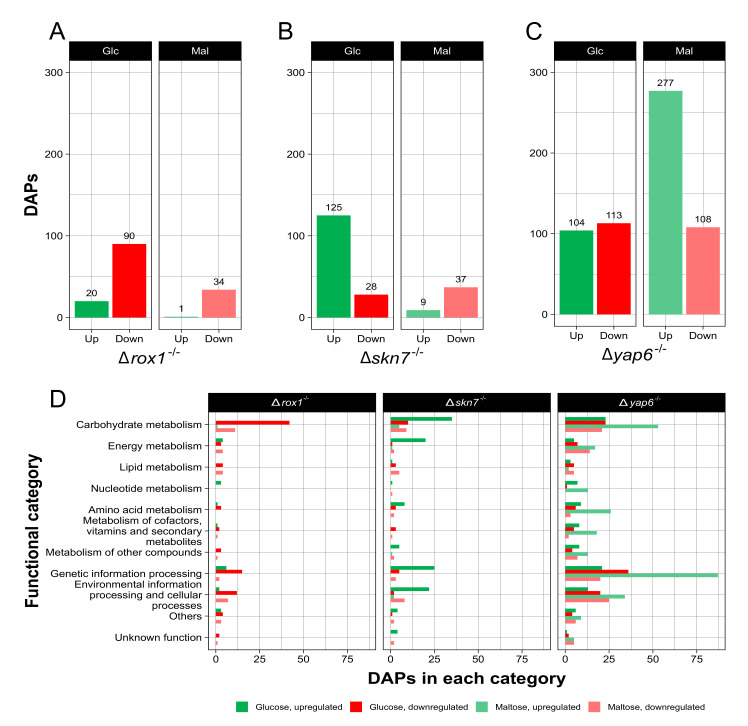
Differentially abundant proteins (DAPs) of Δ*rox1*^−/−^, Δ*skn7*^−/−^, and Δ*yap6*^−/−^ strains in *X. dendrorhous*. Mutant strains grown in glucose or maltose were compared with the wild-type strain grown in glucose or maltose, respectively. Data representing DAPs whose levels were increased or decreased by maltose or glucose treatment. DAPs were determined by FDR *p* value ≤ 0.05. The bar plots show the total DAPs identified for the Δ*rox1*^−/−^ (**A**), Δ*skn7*^−/−^ (**B**), and Δ*yap6*^−/−^ (**C**) strains in each condition. The horizontal bar plot shows the total DAPs in each functional category based on ortholog annotation in the KEGG public database (**D**). Upregulated DAPs (green for glucose, light green for maltose) indicate increased levels in the mutant strain, while downregulated DAPs (red for glucose, light red for maltose) indicate decreased levels in the mutant strain.

To study the effect of the deletion of these transcription factors on the proteome, iTRAQ analyses were performed on the wild-type and Δ*rox1*^−/−^, Δ*skn7*^−/−^, and Δ*yap6*^−/−^ strains in both carbon sources as described for RNA-seq. Each comparison was performed between a mutant strain and the wild-type strain in the same carbon source. Thus, 1480 different proteins were identified. In those experiments, it was observed that 10.7% of the total proteins identified were classified as differentially abundant proteins (DAPs). Interestingly, more DAPs were identified under glucose than under maltose treatment for the Δ*rox1*^−/−^ and Δ*skn7*^−/−^ strains (Figure 4A,B), while the number of DAPs was notably higher under maltose treatment for the Δ*yap6*^−/−^ strain (Figure 4C).

For the Δ*rox1*^−/−^ strain, 20 and 90 DAPs were upregulated and downregulated under glucose conditions, respectively. Additionally, 1 and 34 DAPs were upregulated and downregulated in maltose, respectively (Figure 4A). These results showed a tendency of DAPs to be downregulated in this mutant strain under both growth conditions, which was not observed in the other mutant strains.

For the Δ*skn7*^−/−^ mutant strain, 125 and 28 DAPs were upregulated and downregulated in glucose, respectively, while 9 and 37 DAPs were upregulated and downregulated in maltose, respectively (Figure 4B). Notably, there was a fourfold increase in upregulated DAPs in glucose compared to downregulated DAPs, while in maltose, the upregulated DAPs were a quarter of downregulated DAPs under the same conditions.

In the case of the Δ*yap6*^−/−^ strain, 104 and 113 DAPs were upregulated and downregulated under glucose conditions, respectively. For maltose, 277 DAPs were upregulated, while 108 were downregulated (Figure 4C). It is remarkable that the total DAPs upregulated in maltose were at least double the DAPs upregulated in glucose.

When DAPs identified between all strains and conditions were compared, two DAPs were found to be downregulated, which correspond to the alpha and beta subunits of the fatty acid synthase complex (A0A0F7SEZ3, A0A0F7SU12). Concerning DAPs shared by growth conditions, 22 DAPs were observed in glucose for all strains, where almost all were downregulated in the Δ*rox1*^−/−^ and Δ*yap6*^−/−^ strains but upregulated in the Δ*skn7*^−/−^ strain. Under maltose conditions, 13 common DAPs were observed, and all were downregulated, except for an alcohol dehydrogenase protein (ADH) in the Δ*yap6*^−/−^ strain.

DAPs were classified into functional categories as before (Figure 4D). The “Carbohydrate metabolism” category was the most represented among all DAPs in the Δ*rox1*^−/−^ and Δ*skn7*^−/−^ strains. For Δ*rox1*^−/−^, where mainly downregulated DAPs were found, DAPs mostly belonged to glycolysis and sugar metabolism. Notably, for the Δ*skn7*^−/−^ strain, almost half of the DAPs in glucose were classified as “Energy metabolism,” and nearly 25% of the DAPs were related to “Lipid metabolism” when maltose was the sole carbon source. Upregulated DAPs in glucose were principally classified into “Carbohydrate metabolism” (glycolysis, TCA cycle, and sugar metabolism), “Genetic information processing” and “Energy metabolism.” For downregulated DAPs in maltose, glycolysis, pyruvate, and sugar metabolism were found to be more represented. Finally, for the Δ*yap6*^−/−^ strain, DAPs were mainly involved in glycolysis, the TCA cycle, pyruvate, and the glyoxylate pathway. Interestingly, an increase in total upregulated DAPs related to “Genetic information processing” and “Carbohydrate metabolism” was observed under maltose conditions, which agreed with the increase in maltose-upregulated DAPs mentioned before.

### 2.4. Integrative Metabolic Analysis of X. dendrorhous under the Effect of Deletion of Transcription Factors

To study the global effect of the deletion of each transcription factor, data from phenotypic, transcriptomic, and proteomic analyses were correlated in each condition studied. The growth of the different strains was evidently slower in maltose than glucose, taking a longer time to reach the exponential growth phase (Figure 2A,E). However, under maltose conditions, a higher production of mycosporines was obtained for the wild-type and Δ*skn7*^−/−^ strains compared to the glucose medium (Figure 2C,G). It is noteworthy that a higher increment in mycosporine production was achieved for Δ*skn7*^−/−^ than for the wild-type strain when both carbon sources were compared. Regarding carotenoid production, no significant differences were found in the medium with maltose between strains, except for Δ*yap6*^−/−^ strain (Figure 2F). However, in glucose, the Δ*rox1*^−/−^ strain showed significantly lower pigment production (Figure 2B). Sterol production showed differences between the two carbon sources in each strain except for the Δ*skn7*^−/−^ strain (Figure 2D,H). Notably, the highest sterol production was observed for the Δ*yap6*^−/−^ strain in glucose medium (Figure 2D). As a tendency, in glucose medium, we observed more sterol production than in maltose for Δ*rox1*^−/−^, Δ*yap6*^−/−^, and wild-type strains (Figure 2D,H).

For each mutant strain, modular metabolic analyses were carried out based on the DEGs and DAPs identified. The Δ*rox1*^−/−^ strain showed significant downregulation in carbohydrate metabolism, mainly in glycolysis/gluconeogenesis, the PP pathway, and metabolism of sugars such as starch, sucrose, trehalose, lipid metabolism, and fatty acid synthesis under glucose conditions. This strain showed lower mycosporine production than the wild-type strain in glucose. In maltose, a significant upregulation was observed in carbohydrate metabolism, mainly in glycolysis/gluconeogenesis, the nonoxidative PP pathway, and downregulation of lipid metabolism, specifically in the synthesis of fatty acids and unsaturated fatty acids. Notably, the Δ*rox1*^−/−^ strain produced less sterol than the wild-type strain under this condition.

In the case of the Δ*skn7*^−/−^ strain in glucose, modular metabolic analysis showed significant upregulation in carbohydrate metabolism, mainly in glycolysis/gluconeogenesis, the TCA cycle, oxidative phosphorylation, and sugar and amino acid metabolism, and presented downregulation of lipid metabolism, specifically in fatty acid synthesis. As mentioned before, this strain exhibits the lowest sterol production under this condition. When the Δ*skn7*^−/−^ strain grows in medium with maltose, it produces more mycosporines than the other mutant strains. Once again, the metabolic modular analysis showed significant upregulation in glycolysis/gluconeogenesis, in the reductive PP pathway, and a downregulation of lipid metabolism, specifically in the synthesis of fatty acids and unsaturated fatty acids.

As mentioned before, the Δ*yap6*^−/−^ strain showed a longer lag phase in both carbon sources. In glucose, this strain produces significantly more total carotenoids and sterols than the other strains. Modular metabolic analysis showed significant upregulation in carbohydrate metabolism, glycolysis/gluconeogenesis, and pentose and glucuronate interconversion. Under maltose conditions, this strain produces significantly fewer mycosporines and more carotenoids and sterols than the other strains. Modular metabolic analysis showed significant upregulation in carbohydrate metabolism, glycolysis/gluconeogenesis, the PP pathway, and other sugar metabolism pathways.

The k-numbers obtained for DEGs and DAPs were mapped in the KEGG reference pathway. Figure S1 shows a detailed dataset analysis of the carbon metabolism of each mutant in response to both carbon sources. For the Δ*rox1*^−/−^ strain in glucose, clear downregulation at the protein level prevailed, which was related to glycolysis (M00001), the nonoxidative (M00007) and reductive PP pathways (M00166), glyoxylate (M00012), and the TCA cycle (M00009). However, in maltose the principal effect is opposite to that observed in glucose and evidenced at the transcript level. Additionally, in the oxidative PP pathway (M00006) the malic enzyme NAD was downregulated at the transcript level (Figure S1A,B).

For the Δ*skn7*^−/−^ strain in glucose, there was an observable upregulation effect in DEGs and DAPs related to the nonoxidative PP pathway (M00007), pyruvate oxidation (M00307), and second carbon oxidation of the TCA cycle (M00011). Downregulation is present in the reductive PP pathway (M00166) and acetyl-CoA generation through acetate. The same strain treated with maltose affected transcripts; in contrast, the nonoxidative PP pathway (M00007) was downregulated. Similar behavior shows glycolysis (M00001), the oxidative PP pathway (M00006), and acetyl-CoA generation through acetate. The incomplete reductive TCA cycle (M00620) is upregulated at the transcript level (Figure S1C,D).

The Δ*yap6*^−/−^ strain showed a mixed effect of upregulation and downregulation on glucose and maltose. Glycolysis (M00001) and pyruvate oxidation (M00307) were upregulated in both conditions, except for the step from fructose-1,6-biphosphate to glyceraldehyde-triphosphate, and from phosphoenolpyruvate (PEP) to pyruvate (Pyr), which were downregulated. The first step connects glycolysis to the PP pathway, which is downregulated for the reductive (M00165) and partial oxidative (M00006) pathways. Notably, the nonoxidative phase of the PP pathway was downregulated by maltose (Figure S1E,F). Under both conditions, cysteine biosynthesis (M00021), the C4-dicarboxylic acid cycle, and phosphoenolpyruvate carboxykinase type (M00170) were upregulated. Similarly, the C4-dicarboxylic acid cycle and NADP-malic enzyme type (M00171) from PEP to oxaloacetate were upregulated. However, in the same pathway, the step from pyruvate to malate was downregulated. Fatty acid synthesis using acetyl-CoA (RM018) was upregulated until acetoacetyl-CoA generation. This step is a key intermediary between fatty acid synthesis/beta-oxidation and the beginning of the mevalonate pathway to generate sterols or carotenoids in this yeast (Figure S1E,F). Finally, the upregulation of 2-oxocarboxylic acid chain extension by the tricarboxylic acid pathway (RM001), the generation of acetyl-CoA from acetate, the TCA cycle (M00009), the reductive TCA cycle (M00173), the glyoxylate cycle (M00012), and PRPP biosynthesis (M00005) were observed only under maltose treatment (Figure S1F).

## 3. Discussion

*X. dendrorhous* can grow utilizing different carbon sources; it prefers fermentation over respiration, and its carotenoid production is a special adaptation characteristic, which motivated the study of its phenotypic plasticity. In this work, we combined comparative transcriptomic and proteomic analyses to study the effect of the deletion of stress-related transcription factors and correlate them with phenotypic characteristics. For carotenogenic yeast, this work represents the first approximation to study Δ*rox1^−/−^,* Δ*skn7^−/−^*, and Δ*yap6^−/−^* mutant strains under two fermentable carbon sources.

### 3.1. A Moderate Metabolic Effect Related to the Carbon Source over Mutant Strains

In *X. dendrorhous*, the carbon source is a determinant not only of growth, basal metabolism, and astaxanthin production, but also of regulation at different levels, which is not restricted to transcriptional activation or repression. For instance, in *S. cerevisiae*, glucose represses genes associated with mitochondrial function, gluconeogenesis, and metabolism of other carbon sources during catabolic repression [30]. The effect of glucose is extended to increase the rate of protein degradation, a process known as catabolite inactivation [31]. Looking for the classical proteins regulated by catabolic repression, the glyoxylate enzymes isocitrate lyase (ICL) and malate synthase (MLS) were found to be upregulated at the transcriptomic level and downregulated at the proteomic level in the *Δrox1^−/−^* and Δ*skn7*^−/−^ strains in maltose. In the Δ*yap6^−/−^* strain, both the transcripts and proteins were upregulated. In addition, the gluconeogenesis enzymes fructose 1,6-bisphosphatase (FBP) and malate dehydrogenase (MDH) were both upregulated at the transcriptomic level in this strain (Figure S1). Some mitochondrial proteins were upregulated at the transcript level in the Δ*rox1^−/−^* and Δ*yap6^−/−^* mutant strains, such as aconitase, cytochrome c oxidase, and NADH dehydrogenase. The same behavior was observed for HSP90, HSP10, and HSP20 at the transcript level for all strains studied. These observations were opposite to previous reports in *S. cerevisiae* related to alternative carbon source consumption [32,33]. Differential upregulation of ribosomal proteins, translation factors, and plasma membrane ATPase proteins were not observed under glucose or maltose growth conditions.

Maltose is a fermentable disaccharide composed of two glucose units joined by an α (1→4) bond. In *S. cerevisiae*, maltose is transported through maltose permease (MALT) and cleaved intracellularly into two units of glucose by maltase (MALS). The expression of both enzymes is induced by maltose and repressed by glucose, and regulation occurs predominantly at the transcriptional level by the factor MIG1 [34]. In the comparisons made, unfortunately, genes related to transport or assimilation of maltose were not found in DEGs or DAPs. Only maltose transacetylase, an enzyme that transfers the acetyl group of acetyl-coenzyme A to maltose, was found to be upregulated in maltose at the transcriptomic level.

### 3.2. Phenotypic and Metabolic Profile of Mutant Strains

Based on our results, several metabolic effects were observed between the Δ*rox1^−/−^*, Δ*skn7^−/−^*, and Δ*yap6^−/−^* mutant strains of *X. dendrorhous*.

ROX1 belongs to a highly conserved transcription factor family characterized by a nuclear HMG-box domain. In conjunction with the corepressor complex CYC8–TUP1, the repressor ROX1 avoids the expression of hypoxic genes in the presence of oxygen and helps to preserve cellular and mitochondrial integrity [35]. The repression depends on ROX1 binding to the regulatory region of specific hypoxic genes and the presence or absence of binding sites for ROX1 repression. Interestingly, it has been described that ROX1 inhibits genes related to mevalonate and sterol biosynthesis in *S. cerevisiae* [36,37]. In the present study, the Δ*rox1^−/−^* strain showed a significant reduction in sterol, carotenoid, and mycosporine production (Figure 2). In fact, this strain presented a clear effect of downregulation at the protein level (Figure 4A). Additionally, the upregulation of proteins related to glycolysis, sterol biosynthesis, the PP pathway, the TCA cycle, and the synthesis of some amino acids was observed. Some enzymes related to terpenoid synthesis were upregulated, but carotenogenesis-specific enzymes were downregulated, which is correlated with the low pigment production observed under glucose conditions.

*X. dendrorhous* is an oleaginous yeast [38] that consumes large quantities of ATP and NADPH during lipid and carotenoid synthesis. The cellular sources of NADPH include several pathways, such as the PP pathway, the malic enzyme (ME) transhydrogenase cycle, or the cytoplasmatic NADP+-dependent isocitrate dehydrogenase [39].

The yeast activator (AP1) protein family in *S. cerevisiae* includes eight members (YAP1 to YAP8). YAP4 and YAP6 interact with the transcriptional repressor TUP1, and they probably act as transcriptional repressors in the osmotic stress response [22]. YAP6 and YAP4 share targets related to oxidoreductase activity, hexose transport, and glucose and ethanol catabolism. Specifically, YAP6 target genes encode ribosomal proteins [27]. In *X. dendrorhous*, the Δ*yap6^−/−^* strain grew slowly and produced more sterols and pigments than any other of the strains studied. In a metabolic context, it is related to an upregulation of the step to form acetyl-CoA from pyruvate, acetate, and acetoacetyl-CoA (Figure S1E,F). Acetyl-CoA performs important roles at the cellular level, for instance, at the beginning of the TCA cycle, and acts as a precursor for the biosynthesis of sterols, fatty acids, lipids, amino acids, and polyketides [39]. The two major acetyl-CoA-derived biosynthetic pathways in *X. dendrorhous* are terpenoid and fatty acid biosynthesis. Indeed, the terpenoid pathway leads to the synthesis of sterols and carotenoids in this yeast [40]. Acetoacetyl-CoA is one intermediary of mevalonate synthesis, and this pathway is involved in sterol and carotenoid biosynthesis. In the Δ*yap6^−/−^* strain, we found that acetoacetyl-CoA formation by acetoacetyl-CoA thiolase was upregulated at the transcriptomic level under both growth conditions (Figure S1). Other mevalonate enzymes, such as hydroxymethylglutaryl synthase (HMG-S) and isopentenyl diphosphate isomerase (IDI), were also upregulated in this strain. The mevalonate pathway is in competition with fatty acid biosynthesis [39]. Similarly, enzymes related to fatty acid synthesis were significantly downregulated in all strains. The carotenoid enzymes geranylgeranyl pyrophosphate synthase (*crtE*) and astaxanthin synthase (*crtS*) were upregulated in Δ*yap6^−/−^* in both carbon sources, while phytoene-β-carotene synthase (*crtYB*) was upregulated only under maltose treatment. These results correlate with the higher carotenoid production in this strain in glucose. On the other hand, the PP pathway was downregulated in maltose, which correlated with the lowest production of mycosporines between all strains studied. Another interesting finding was that phosphoketolase was significantly upregulated in maltose medium at the transcriptomic level. This enzyme normally enables higher efficiency of carbon metabolism since it bypasses the wasteful decarboxylation of pyruvate to acetyl-CoA [39]. This suggests an active formation of acetyl-CoA from pyruvate, which is related to the increased availability of precursors for sterol biosynthesis and carotenogenesis. Additionally, downregulation of the PP pathway would act as an annex way to regenerate NADPH. Finally, the aldehyde dehydrogenase was significantly upregulated in maltose at the transcript level. In fact, in our previous works [12,20], we found a prevalent upregulation of this enzyme in the wild-type strain upon treatment with alternative carbon sources, such as succinate and maltose, and in the CYC8, TUP1, and MIG1 mutant strains. Indeed, as described in oleaginous yeast [39], this could be related to another potential source of NADPH in this yeast.

In *S. cerevisiae*, the transcription factors YAP1 and SKN7 have a stress-responsive role through a specific and cooperative control of two gene subsets, the antioxidant scavenging enzymes and the metabolic pathways to regenerate the main cellular reducing power, such as glutathione and NADPH [41]. A previous screening showed that a SKN7-dependent subset of YAP1-controlled genes are important for peroxide tolerance, such as thioredoxin (TRX2) and thioredoxin reductase (TRR1), which includes many enzymes related to carbohydrate metabolism, amino acid metabolism, and a few heat shock proteins, among others [41,42]. In accordance, we found that for the Δ*skn7^−/−^* strain, TRR1, thioredoxin peroxidase, HSP70, and HSP60 were upregulated in both carbon sources, and glutathione s-transferase was downregulated. In contrast, MnSOD was upregulated only in glucose. Catalase, HSP30, transaldolase, and glucose-6-phosphate 1-dehydrogenase were downregulated in maltose. Therefore, this could indicate that the YAP1 protein in *X*. *dendrorhous* overcomes the absence of SKN7 to regulate several target genes, but it is not enough or specific to regulate catalase and some PP pathway enzymes, such as transaldolase.

In reference to the Δ*skn7^−/−^* strain, it is noteworthy that this mutant was the one that produced more mycosporines in maltose and the one that produced fewer sterols than the wild-type strain in glucose. For *X. dendrorhous*, carotenoid and ergosterol synthesis are exclusively generated through the mevalonate pathway [43] with acetyl-CoA as the principal precursor, and it requires ATP and NADPH. Therefore, these pathways are in competition with fatty acid biosynthesis [39]. Mycosporine biosynthesis has been less studied, but it has been proposed that it is related to the shikimic acid pathway, which uses 3-dehydroquinate (DHQ) as a precursor [44]. However, an alternative biosynthetic pathway has been suggested that uses sedoheptulose 7-phosphate (S7P) as a precursor [45]. S7P is an intermediate of the PP pathway. To produce shinorine, a mycosporine-derived compound, S7P is converted to 4-deoxygadusol (4-DG) by 2-demethyl-4-deoxygadusol synthase (DDGS) and O-methyltransferase (O-MT). Then, glycine is conjugated to 4-DG by an ATP-grasp enzyme to form mycosporine-glycine (MG), which is the shinorine precursor [45]. We have identified a gene cluster that encodes DDGS, OMT, and ATP-grasp in the genome of *X. dendrorhous* [43]. In the transcriptomic analysis of the Δ*skn7^−/−^* strain, ATP-grasp was upregulated in both carbon sources (Table S2). Looking to the related pathways, S7P and erythrose-4P are generated by the nonoxidative PP pathway. For glucose, this pathway is upregulated at the protein level, and in contrast, for maltose, it is downregulated at the transcriptomic level (Figure S1C,D). The aromatic amino acid biosynthesis pathway was upregulated at the transcriptomic level in both carbon sources (Table S2). Glycolysis is downregulated at the transcriptomic level only by maltose. In summary, there was low sterol production in the Δ*skn7^−/−^* strain in glucose, which may be related to active oxidative metabolism and upregulated amino acid synthesis. For maltose, there is more active synthesis of mycosporines. This metabolic difference could be a response to the lack of SKN7 and the offset regulation by YAP1 in *X. dendrorhous*, in addition to the imbalance of other functions associated with SKN7, such as cell wall biosynthesis control, the cell cycle, and the osmotic stress response [41].

Altogether, these results showed that carotenoids and mycosporines are regulated by stress-related transcription factors such as ROX1, SKN7, and YAP6 in *X. dendrorhous*. These secondary metabolites may be part of the adaptative response to the environment of this yeast, and its biosynthesis would be activated when yeast faces unfavorable growth conditions by directly affecting the transcription of their biosynthetic genes or regulating the total carbon efflux to their related metabolic pathways. Since these transcription factors interact with the CYC8–TUP1 complex in other organisms and the CYC8–TUP1 complex is involved in astaxanthin regulation in *X. dendrorhous*, this complex becomes a fundamental piece in modulating the yeast response to its environment through secondary metabolites, which is not restricted to carbon catabolic repression but could also be involved in the regulation of several other biological processes.

## 4. Materials and Methods

### 4.1. Strains and Culture Conditions

*X. dendrorhous* strains (see Table S1A in the Supplemental Material) were grown at 22 °C with constant shaking in a 1 L Erlenmeyer flask containing 380 mL of 0.7% YNB minimal medium (YNB DIFCO BD 291940) supplemented either with 2% glucose or 2% maltose, the carbon source was employed as previously described [20]. The cells were grown to the early exponential growth phase (OD600 of 1.6–2.5) and then collected for phenotypical characterization, transcriptomics, and proteomics experiments. Optical density was measured at 600 nm using a JASCO V-630 spectrophotometer (JASCO Inc., Easton, MD, USA). Samples were harvested through centrifugation, and the pellet was washed twice with ice-cold water, centrifuged at 5000× *g* for 10 min at 4 °C, and stored at −80 °C until further analysis.

*X. dendrorhous* mutant strains for the genes *ROX1, SKN7,* or *YAP6* were derived from the wild-type strain UCD 67–385 (ATCC 24230) and were obtained via homologous recombination to replace the coding region (from the ATG to the stop codon) of the corresponding gene with a cassette that confers resistance to an antibiotic [2] (see Table S1A–D and Figure S2).

The *Escherichia coli* strains were cultured with constant shaking at 37 °C in lysogeny broth medium. The agar plate-lysogeny broth was supplemented with 100 μg/mL ampicillin and 32 μg/mL X-gal (5-bromo-4-chloro-3-indolyl-β-D-galactopyranoside) for the selection of recombinant clones [2].

### 4.2. Identification of ROX1, YAP6, and SKN7 Genes in X. dendrorhous

The *ROX1, SKN7,* and *YAP6* genes of *X. dendrorhous* were identified through homology searches using blastp [46]. Sequences Rox1p (NP_015390), Skn7p (NP_012076), and Yap6p (NP_010545) from *S. cerevisiae* were searched against the transcriptome of *X. dendrorhous* available in our laboratory [47]. Transcripts with the best e-value were selected as orthologs of each gene. Graphical representations of genes and gene products were carried out on IBS 1.0.3 software [48]. Conserved domain analysis was carried out with CDD database v3.19 [49], with an e-value threshold = 0.01.

### 4.3. Phenotypic Determination

#### 4.3.1. Pigment Extraction

Pigments were extracted from cellular pellets by acetone extraction [50]. In brief, aliquots of cell cultures were collected under the different conditions tested. The aliquots were centrifuged at 4000× *g*, and the supernatants were subsequently discarded. Each cell pellet was suspended in 2 mL of an acetone:water mixture (1:1), and 500 μL of 0.5 mm glass beads was then added. After 3 min of vortex shaking, the mixture was centrifuged at 4000× *g* for 5 min. Next, the supernatant was transferred to a clean test tube, and 2 mL of acetone was added to the pellet. The tube containing the pellet was then vortexed, stirred for 3 min and centrifuged at 4000× *g* for 3 min, after which the supernatant was collected and mixed with the supernatant that had been previously set aside. These steps were repeated until the recovered supernatant was completely colorless.

The collected supernatants were then treated with 0.25 volumes of water and 0.25 volumes of petroleum ether; this mixture was mixed and centrifuged for 5 min at 4000× *g*. Subsequently, the petroleum ether (top) phase was recovered, and its absorbance at 474 nm using an absorption coefficient of A1% = 2100 was determined and normalized to the yeast dry weight. The analyses were performed in triplicate.

#### 4.3.2. Sterol Extraction

The sterol extraction was modified from a previous work [51]. Briefly, a cell pellet from 15 mL of culture was mixed with 4 g of KOH and 16 mL of 60% ethanol solution (*v*/*v*). For saponification, the mixture was incubated at 80 °C in a water bath for 2 h. Then, after the mixture was cooled, sterols were extracted with 5 mL of petroleum ether vortexed for 10 s and centrifuged for 5 min. The concentration of sterols was spectrophotometrically determined at 280 nm using the molar extinction coefficient E_m_ = 11,900 M^−1^ cm^−1^ and normalized by the dry weight of the yeast.

#### 4.3.3. Mycosporine Extraction

For the mycosporine extraction, the cell pellets were resuspended in 1 mL of 80% ethanol, incubated for 2 h at 80 °C, and then centrifuged at 14,000 rpm for 2 min. In the recovered supernatant, the mycosporine concentration was spectrophotometrically determined at 310 nm using the molar extinction coefficient E_m_ = 25,000 M^−1^ cm^−1^ [52] and normalized by the dry weight of the yeast.

### 4.4. Statistical Analyses

One-way ANOVA followed by Fisher’s LSD test on phenotypic data was performed using GraphPad Prism version 9.3.1 for Windows, GraphPad Software, San Diego, CA, USA, www.graphpad.com (accessed on 18 March 2022). No mathematical correction was made for multiple comparisons, and all comparisons performed were planned and reported in this work.

#### 4.4.1. RNA Extraction, Transcriptome Sequencing, and RNA-Seq Analysis

RNA extraction was performed according to a previously described protocol [53]. RNA sequencing was performed by Macrogen Inc. (Seoul, Korea) using the TruSeq Stranded mRNA LT Sample Prep Kit on the Illumina platform with a 100 bp paired-end read length. Raw transcriptomic data were analyzed with CLC Genomics Workbench 20.0.2 for quality control and mapping against the transcriptome of *X. dendrorhous* UCD 67–385 available in our laboratory. Differential expression analysis was carried out using DESeq2 version 1.26.0 [54] in R 3.6.1, which are available in the Bioconductor repository (www.bioconductor.org, accessed on 18 March 2022) and in the R-project (www.r-project.org, accessed on 18 March 2022), respectively. In this work, transcripts with an absolute log2-fold-change ratio > 1 and adjusted *p* value ≤ 0.05 were considered DEGs, as previously described [20]. Adjusted *p* values were estimated using the Benjamini and Yekutieli FDR method [55], which is implemented in DESeq2 software. Comparisons were carried out between a mutant strain and wild-type strain in the same carbon source. The transcriptome quantitation information is provided in Table S2.

#### 4.4.2. Protein Extraction, Digestion, and Labeling with iTRAQ Reagents

Proteins were extracted as previously described [20]. Briefly, each cell pellet was treated with 1 volume of lysis buffer (100 mM sodium bicarbonate, 0.5% Triton X100, 1 Mm PMSF, 2% protease inhibition cocktail (Promega, Madison, WI, USA), 2 mM TCEP), and glass beads (0.5 mm). Seven cycles of disruption of 30 s each were performed using the “cell grinder” Mini-Beadbeater-16 (Biospec, Bartlesville, OK, USA). Between each disruption cycle, the samples were incubated on ice for 1 min. Then, centrifugation was performed at 4 °C for 20 min at 14,000 rpm, and the supernatant was recovered. The supernatants were incubated for 30 min at room temperature in a 10% *v*/*v* DNase-RNase solution (0.5 M Tris–HCl, pH 7.0, 0.5 M MgCl_2_, 100 μg/mL RNase A; Boehringer Mannheim, Germany) containing 2 μL DNase I (Boehringer Mannheim), and the final volume was adjusted to 2.5 mL with deionized water. The proteins were analyzed in acrylamide gels under denaturant conditions (SDS-PAGE) and quantified using a Pierce^®^ BCA Protein Assay Kit (Thermo Scientific, Waltham, MA, USA). The protein extracts obtained from three biological replicates (different independent cultures) were stored at −80 °C.

Proteomics analyses were performed in the National Center of Biotechnology (CNB-CSIC; Madrid, Spain). For digestion, 50 µg of protein from each condition was precipitated by the methanol/chloroform method. Protein pellets were resuspended and denatured in 20 µL 7 M urea/2 M thiourea, 0.1 M TEAB, pH 7.5, (SERVA Electrophoresis GmbH, Heildelberg, Germany), reduced with 1 µL of 50 mM Tris (2-carboxyethyl) phosphine (TCEP, AB SCIEX), pH 8.0, at 37 °C for 60 min, and followed by 2 µL of 200 mM cysteine-blocking reagent (methyl methanethiosulfonate; MMTS, Pierce) for 10 min at room temperature. Samples were diluted up to 120 µL to reduce the urea/thiourea concentration with 50 mM TEAB.

For digestion of each sample, 2 µg of sequence grade-modified trypsin (Thermo) was added to each sample at a ratio of 1/25 (*w*/*w*), processed at 37 °C overnight on a shaker, and finally evaporated to dryness. Samples were labeled with iTRAQ tags (AB Sciex, Foster City, CA, USA) as follows: iTRAQ 113 reagent = 385_rep1; iTRAQ 114 reagent: 385_rep2; iTRAQ 115 reagent: ROX_rep1; iTRAQ 116 reagent: ROX_rep2; iTRAQ 117: SKN7_rep1; iTRAQ 118 reagent: SKN7_rep2; iTRAQ 119 reagent: YAP6_rep1; iTRAQ 121 reagent: YAP6_rep2. After labeling, the samples were combined, and the labeling reaction was stopped by evaporation in a Speed Vac. Salts were washed using a C18 cartridge (BondElut, Agilent, Santa Clara, CA, USA).

#### 4.4.3. Liquid Chromatography and Mass Spectrometric Analysis

Before LC-MS analysis, the combined sample was prefractionated using high pH reversed-phase spin columns (Pierce). Four independent fractions were obtained (using 7.5%, 12.5%, 20%, and 50% acetonitrile in water and 0.1% triethylamine). A 2 µg aliquot of each fraction was subjected to 2D-nano LC ESI-MS analysis using a nano liquid chromatography system (Eksigent Technologies nanoLC Ultra 1D plus, AB SCIEX, Foster City, CA, USA) coupled to a high-speed Triple TOF 5600 mass spectrometer (SCIEX, Foster City, CA, USA) with a Nanospray III source. The injection volume was 5 µL. The analytical column used was a silica-based reversed-phase column nanoACQUITY UPLC 75 µm × 15 cm (Waters, Milford, MA, USA), 1.7 µm particle size. The trap column was an Acclaim PepMap 100 (ThermoFisher Scientific, Waltham, MA, USA), 5 µm particle diameter, 100 Å pore size, switched online with the analytical column. The loading pump delivered a solution of 0.1% formic acid in water at 2 µL/min. The nanopump provided a flow rate of 250 nL/min and was operated under gradient elution conditions, using 0.1% formic acid in water as mobile phase A and 0.1% formic acid in acetonitrile as mobile phase B. Gradient elution was performed according to the following scheme: isocratic conditions of 96% A: 4% B for five minutes, a linear increase to 40% B in 105 min, then a linear increase to 90% B for 15 additional minutes, isocratic conditions of 90% B for ten minutes, and return to initial conditions in 2 min. The total gradient length was 150 min.

Data acquisition was performed with a TripleTOF 5600 System. All data were acquired using information-dependent acquisition (IDA) mode with Analyst TF 1.7 software (AB SCIEX, Framingham, MA, USA). For IDA parameters, a 0.25 s MS survey scan in the mass range of 350–1250 Da was followed by 30 MS/MS scans of 150 ms in the mass range of 100–1500 (total cycle time: 4.5 s). Switching criteria were set to ions greater than the mass-to-charge ratio (*m/z*) 350 and smaller than *m/z* 1250, with a charge state of 2–5 and an abundance threshold of more than 90 counts (cps). Former target ions were excluded for 20 s. An IDA rolling collision energy (CE) parameter script was used to automatically control the CE.

#### 4.4.4. Data Analysis and Statistics

The search and identification of proteins used Peak View v1.2.0.3 (AB SCIEX, Framingham, MA, USA), combined and searched using Mascot Server 2.5.1 (Matrix Science, Boston, MA, USA), OMSSA 2.1.9 (NCBI, Bethesda, MD, USA), X!TANDEM 2013.02.01.1 ((The Global Proteome Machine Organization, www.thegpm.org, accessed on 18 March 2022), and Myrimatch 2.2.140 (Vanderbilt University, Nashville, TN, USA) against a composite target/decoy database built from the *Phaffia rhodozyma* (*Xanthophyllomyces dendrorhous*) protein entries found at UniProt Knowledgebase (January 2020), together with commonly occurring contaminants. The searches were made according to the following parameters: MS precision of 10 ppm, MS/MS of 0.02 Da, until two cleavage sites are lost and isotope error (13C) of 1 occurs; carbamidomethylation of cysteines, oxidation of methionine, pyroglutamic acid from glutamine or glutamic acid at the peptide N-terminus, N-terminal acetylation of proteins, and modification of lysine, tyrosine, and peptide N-terminus with iTRAQ 8-plex reagents [56]. Score distribution models were used to compute a peptide-spectrum match *p* value, and spectra recovered by a false positive detection rate (FDR) ≤ 0.01 (peptide-level) filter were selected for quantitative analysis. Approximately 5% of the signals with the lowest quality were removed prior to further analysis. Differential regulation was measured using linear models2, and statistical significance was measured using q values (FDR). All analyses were conducted using software from Proteobotics (Madrid, Spain).

For comparative purposes, the proteins obtained from the wild-type strain with glucose as the carbon source were used as a reference. For each inferred protein, the peptides were classified according to their abundance in quantitative groups and then normalized by log2. DAPs were defined as proteins that changed their abundance levels with an FDR *p* value ≤ 0.05 with respect to the control group. The protein quantitation information is provided in Table S2.

#### 4.4.5. Functional Classification of Biological Sequences

The identified proteins were classified into functional categories based on Kyoto Encyclopedia of Genes and Genomes via BlastKOALA [57] and kofamKOALA [58]. KEGG mapping was carried out using the KO Database of Molecular Functions (reference hierarchy), and functional classification was performed as described previously [20].

## 5. Conclusions

We employed an omic approach for in-depth evaluation of the effects of ROX1, YAP6, and SKN7 gene deletion in *X. dendrorhous*. Our results showed a direct effect at the metabolic level in null mutants related to central metabolism and secondary metabolite production. This effect can be correlated with the function described for each stress-related transcription factor studied, and its hierarchical interaction level with the corepressor complex CYC8–TUP1. The biosynthesis of astaxanthin and mycosporines constitutes a biological fitness mechanism mediated by the binding of ROX1, SKN7, YAP6, and other transcription factors to specific sequences present in the promoter regions of their genes, recruiting the CYC8–TUP1 complex and interacting with other unknown factors that would allow for yeast to respond to different types of stress by repressing or activating metabolic pathways. Our results contribute to a better understanding of the role of stress-related transcription factors and the effect of the carbon source on secondary metabolite biosynthesis in *X. dendrorhous*, aiming for its optimization and potential use as an industrial source of astaxanthin.

## Data Availability

All relevant data are included in the article and its supplemental files. The sequences of mRNA and genes *ROX1* (OP021065 and OP021066), *SKN7* (OP021067 and OP021068), and *YAP6* (OP021069 and OP021070) have been deposited in GenBank.

## Supplementary Materials

The following supporting information can be downloaded at: https://www.mdpi.com/article/10.3390/ijms23169282/s1. References [59,60] are cited in the supplementary materials.
