# Supplementary material for: Role of ROX1, SKN7, and YAP6 Stress Transcription Factors in the Production of Secondary Metabolites in Xanthophyllomyces dendrorhous
Source: Int J Mol Sci. 2022 Aug 18;23(16):9282. doi: 10.3390/ijms23169282 (PMC9409151; doi:10.3390/ijms23169282)
Supplement: Supplementary file 1 [file ijms-23-09282-s001.zip › Figure S1.pdf]

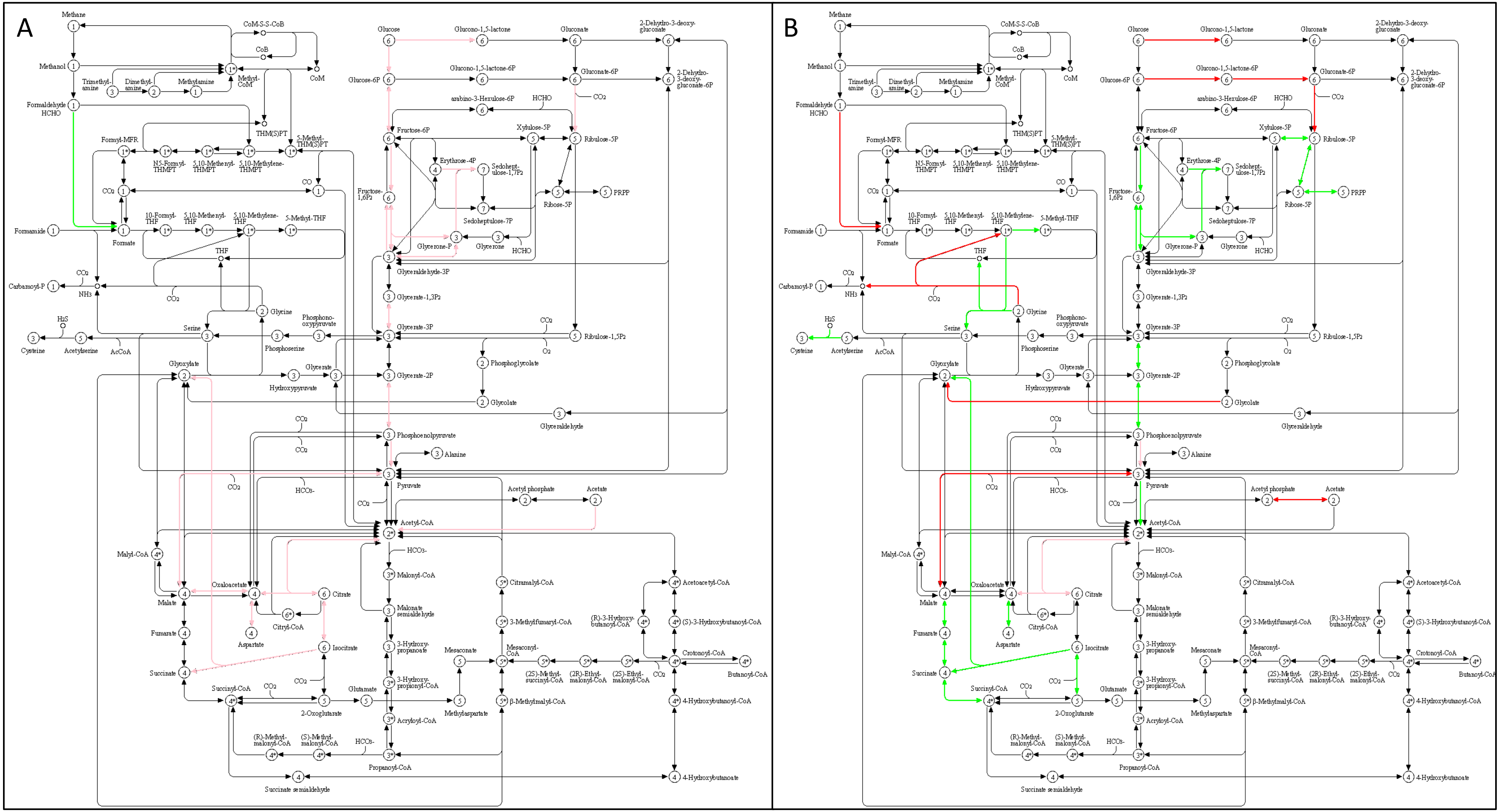
A
B
Methane
CoM-S-S-CoB
CoB
Methanol
CoM
Methyl-CoM
Trimethyl-amine
Dimethyl-amine
Methylamine
Formaldehyde HCHO
THM(S)PT
5-Methyl-THM(S)PT
Formyl-MFR
N5-Formyl-THMPT
5,10-Methenyl-THMPT
5,10-Methylene-THMPT
CO_2
CO
10-Formyl-THF
5,10-Methenyl-THF
5,10-Methylene-THF
5-Methyl-THF
Formamide
Formate
THF
Carbamoyl-P
NH_3
H_2S
Glycine
Phosphono-oxypyruvate
Serine
Cysteine
Acetylserine
AcCoA
Phosphoserine
Glyoxylate
Glycerate
Hydroxypyruvate
Glucose
Glucono-1,5-lactone
Gluconate
2-Dehydro-3-deoxy-gluconate
Glucose-6P
Glucono-1,5-lactone-6P
Gluconate-6P
2-Dehydro-3-deoxy-gluconate-6P
arabino-3-Hexulose-6P
HCHO
Fructose-6P
Xylulose-5P
Ribulose-5P
Erythrose-4P
Sedohept-ulose-1,7P2
Fructose-1,6P2
Ribose-5P
PRPP
Sedoheptulose-7P
Glycerone-P
Glycerone
Glyceraldehyde-3P
Glycerate-1,3P2
Glycerate-3P
Ribulose-1,5P2
O_2
Phosphoglycolate
Glycerate-2P
Glycolate
Glyceraldehyde
Phosphoenolpyruvate
Alanine
Pyruvate
HCO_3^-
Acetyl phosphate
Acetate
Acetyl-CoA
Malonyl-CoA
Malyl-CoA
Citramalyl-CoA
Acetoacetyl-CoA
Oxaloacetate
Citrate
Malate
Citryl-CoA
Malonate semialdehyde
3-Methylfumaryl-CoA
(R)-3-Hydroxy-butanoyl-CoA
(S)-3-Hydroxybutanoyl-CoA
Fumarate
Aspartate
3-Hydroxy-propanoate
Mesaconate
Mesaconyl-CoA
Isocitrate
Crotonoyl-CoA
Butanoyl-CoA
Succinate
3-Hydroxy-propionyl-CoA
(2S)-Methyl-succinyl-CoA
(2R)-Ethyl-malonyl-CoA
(2S)-Ethyl-malonyl-CoA
Succinyl-CoA
Glutamate
2-Oxoglutarate
Methylaspartate
β-Methylmalyl-CoA
4-Hydroxybutanoyl-CoA
Acryloyl-CoA
(R)-Methyl-malonyl-CoA
(S)-Methyl-malonyl-CoA
Propanoyl-CoA
Succinate semialdehyde
4-Hydroxybutanoate

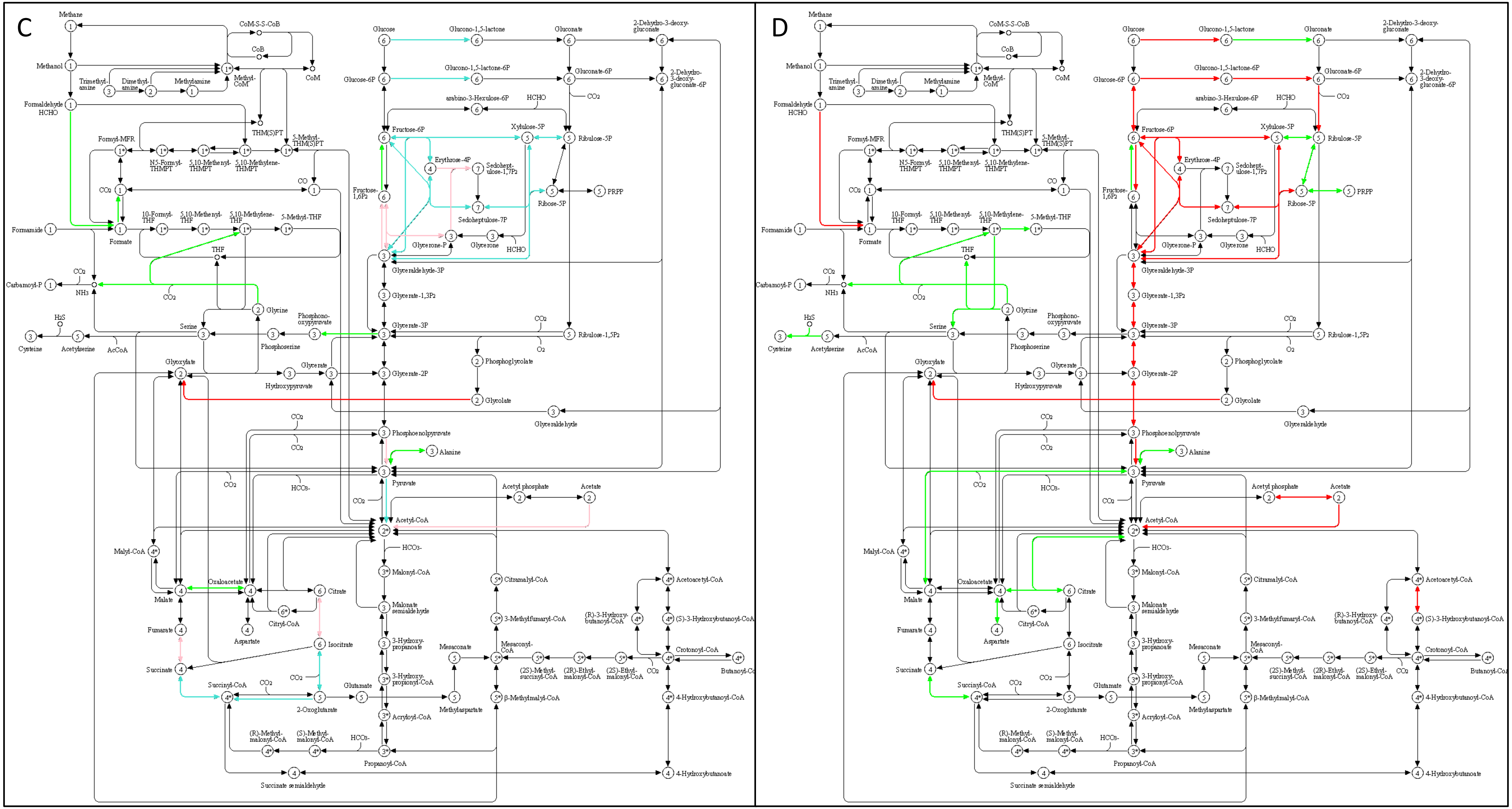
C
D
Methane
Methanol
CoM-S-S-CoB
CoB
CoM
Methyl-CoM
Trimethyl-amine
Dimethyl-amine
Methylamine
Formaldehyde HCHO
THM(S)PT
5-Methyl-THM(S)PT
Formyl-MFR
N5-Formyl-THMPT
5,10-Methenyl-THMPT
5,10-Methylene-THMPT
CO2
CO
Formamide
Formate
10-Formyl-THF
5,10-Methenyl-THF
5,10-Methylene-THF
5-Methyl-THF
THF
Carbamoyl-P
NH3
Glycine
Serine
H2S
Cysteine
Acetylserine
AcCoA
Phosphoserine
Phosphono-oxypyruvate
Glyoxylate
Hydroxypyruvate
Glycerate
Glucose
Glucono-1,5-lactone
Gluconate
2-Dehydro-3-deoxy-gluconate
Glucose-6P
Glucono-1,5-lactone-6P
Gluconate-6P
2-Dehydro-3-deoxy-gluconate-6P
arabino-3-Hexulose-6P
HCHO
Fructose-6P
Xylulose-5P
Ribulose-5P
Erythrose-4P
Sedohept-ulose-1,7P2
Sedoheptulose-7P
Ribose-5P
PRPP
Fructose-1,6P2
Glycerone-P
Glycerone
Glyceraldehyde-3P
Glycerate-1,3P2
Glycerate-3P
Ribulose-1,5P2
O2
Phosphoglycolate
Glycerate-2P
Glycolate
Glyceraldehyde
Phosphoenolpyruvate
Alanine
Pyruvate
HCO3-
Acetyl phosphate
Acetate
Acetyl-CoA
Malyl-CoA
Malonyl-CoA
Malonate semialdehyde
3-Hydroxy-propanoate
3-Hydroxy-propionyl-CoA
Acryloyl-CoA
Propanoyl-CoA
(R)-Methyl-malonyl-CoA
(S)-Methyl-malonyl-CoA
Malate
Oxaloacetate
Citrate
Citryl-CoA
Aspartate
Fumarate
Isocitrate
Succinate
Succinyl-CoA
2-Oxoglutarate
Glutamate
Mesaconate
Methylaspartate
Citramalyl-CoA
3-Methylfumaryl-CoA
Mesaconyl-CoA
β-Methylmalyl-CoA
(2S)-Methyl-succinyl-CoA
(2R)-Ethyl-malonyl-CoA
(2S)-Ethyl-malonyl-CoA
Acetoacetyl-CoA
(R)-3-Hydroxy-butanoyl-CoA
(S)-3-Hydroxybutanoyl-CoA
Crotonoyl-CoA
Butanoyl-CoA
4-Hydroxybutanoyl-CoA
Succinate semialdehyde
4-Hydroxybutanoate

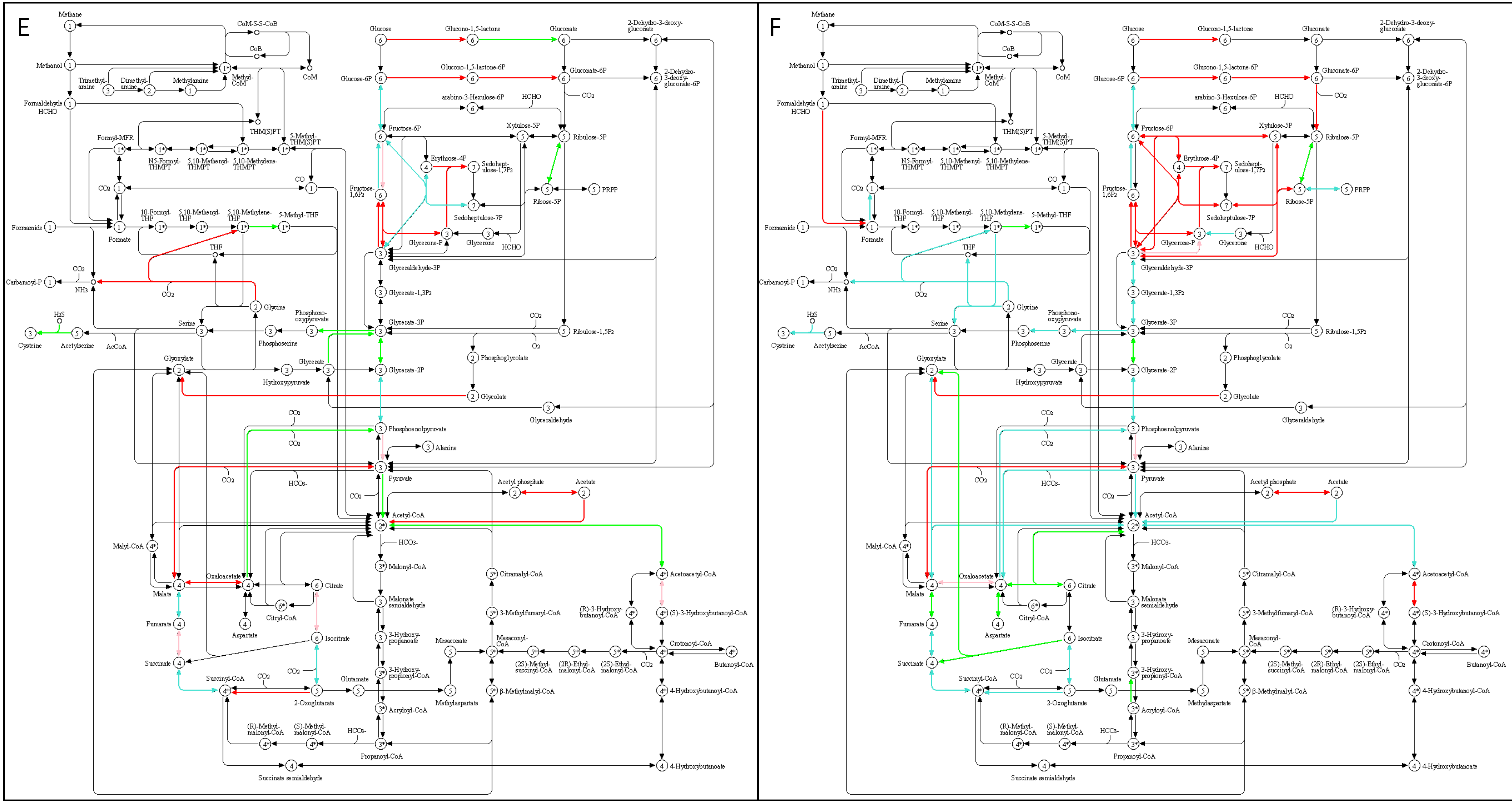
E
Methane
CoM-S-S-CoB
CoB
Methanol
Trimethyl-amine
Dimethyl-amine
Methylamine
Methyl-CoM
CoM
Formaldehyde HCHO
THM(S)PT
5-Methyl-THM(S)PT
Formyl-MFR
N5-Formyl-THMPT
5,10-Methenyl-THMPT
5,10-Methylene-THMPT
CO_2
CO
10-Formyl-THF
5,10-Methenyl-THF
5,10-Methylene-THF
5-Methyl-THF
Formamide
Formate
THF
Carbamoyl-P
NH_3
Glycine
H_2S
Serine
Phosphono-oxypyruvate
Cysteine
Acetylserine
AcCoA
Phosphoserine
Glyoxylate
Glycerate
Hydroxypyruvate
Glucose
Glucono-1,5-lactone
Gluconate
2-Dehydro-3-deoxy-gluconate
Glucono-1,5-lactone-6P
Glucose-6P
Gluconate-6P
2-Dehydro-3-deoxy-gluconate-6P
arabino-3-Hexulose-6P
HCHO
Fructose-6P
Xylulose-5P
Ribulose-5P
Erythrose-4P
Sedohept-ulose-1,7P2
Fructose-1,6P2
Ribose-5P
PRPP
Sedoheptulose-7P
Glycerone-P
Glycerone
Glyceraldehyde-3P
Glycerate-1,3P2
Glycerate-3P
Ribulose-1,5P2
O_2
Phosphoglycolate
Glycerate-2P
Glycolate
Glyceraldehyde
Phosphoenolpyruvate
Alanine
HCO_3^-
Pyruvate
Acetyl phosphate
Acetate
Acetyl-CoA
Malyl-CoA
Malonyl-CoA
Citramalyl-CoA
Acetoacetyl-CoA
Oxaloacetate
Citrate
Malate
Malonate semialdehyde
Citryl-CoA
3-Methylfumaryl-CoA
(R)-3-Hydroxy-butanoyl-CoA
(S)-3-Hydroxybutanoyl-CoA
Fumarate
Aspartate
Isocitrate
3-Hydroxy-propanoate
Mesaconate
Mesaconyl-CoA
Crotonoyl-CoA
Succinate
(2S)-Methyl-succinyl-CoA
(2R)-Ethyl-malonyl-CoA
(2S)-Ethyl-malonyl-CoA
Butanoyl-CoA
Succinyl-CoA
Glutamate
3-Hydroxy-propionyl-CoA
2-Oxoglutarate
Methylaspartate
β-Methylmalyl-CoA
4-Hydroxybutanoyl-CoA
Acryloyl-CoA
(R)-Methyl-malonyl-CoA
(S)-Methyl-malonyl-CoA
Propanoyl-CoA
Succinate semialdehyde
4-Hydroxybutanoate
F

Fig. S1. Carbon metabolism mapping of Δ*rox1*$^{-/-}$, Δ*skn7*$^{-/-}$, and Δ*yap6*$^{-/-}$ strains in *X. dendrorhous*.

DAPs and DEGs identified were mapped to carbon metabolism reference map in KEEG database. Strain Δ*rox1*$^{-/-}$ in glucose (A) or maltose (B); Δ*skn7*$^{-/-}$ in glucose (C) or maltose (D); and Δ*yap6* $^{-/-}$ in glucose (E) or maltose (F). DEGs upregulated are repressented by green line, and downregulated by a red line. DAPs upregulated are repressented by turquoise line and downregulated by pink line.
