# Supplementary material for: Role of ROX1, SKN7, and YAP6 Stress Transcription Factors in the Production of Secondary Metabolites in Xanthophyllomyces dendrorhous
Source: Int J Mol Sci. 2022 Aug 18;23(16):9282. doi: 10.3390/ijms23169282 (PMC9409151; doi:10.3390/ijms23169282)
Supplement: Supplementary file 1 [file ijms-23-09282-s001.zip › Figure S2.pdf]

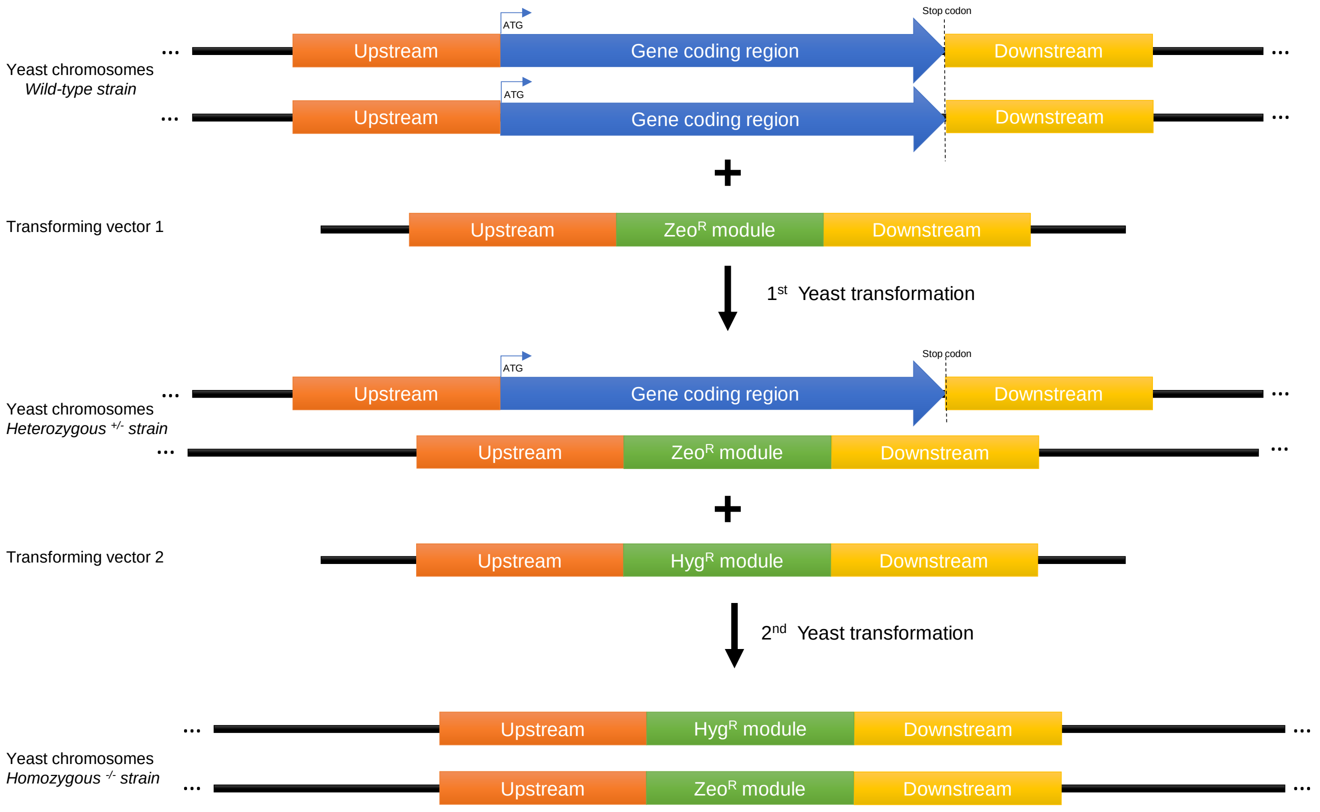

Figure S2. Scheme for mutant construction in *X. dendrorhous*. In this work, the diploid wild-type strain UCD 67-385 was transformed with a zeocin resistance module to obtain the heterozygous transformant by homologous recombination. Then, this strain was transformed with a hygromycin resistance module to obtain the homozygous null mutant Δ*gene*$^{-/-}$, which was able to grow in selective media with both antibiotics. The upstream and downstream regions of target genes were specific for *ROX1*, *SKN7* and *YAP6* in this yeast, and allowed to remove the complete gene coding region.
