# Supplementary material for: Role of ROX1, SKN7, and YAP6 Stress Transcription Factors in the Production of Secondary Metabolites in Xanthophyllomyces dendrorhous
Source: Int J Mol Sci. 2022 Aug 18;23(16):9282. doi: 10.3390/ijms23169282 (PMC9409151; doi:10.3390/ijms23169282)
Supplement: Supplementary file 1 [file ijms-23-09282-s001.zip › Table S1.pdf]

Supplementary Table S1. Material and Methods

A. Yeast strains used in this work

Strain	Description	Reference or source
E. coli DH5α	F- Φ80d lacZΔM15Δ(lacZY-argF)U169 deoR recA1 endA1 hsdR17(rk- mk+) phoA supE44l- thi-1 gyrA96 relA1	[59]
X. dendrorhous UCD 67–385	Diploid wild-type strain (Hyg^S and Zeo^S)	ATCC 24230
Δ*skn7*$^{-/-}$	Homozygous mutant gene *SKN7*_Zeo_hyg (*SKN7*::*ble*/*SKN7*:: *hph*), obtained by transformation of the mutant strain heterozygous gene *SKN7* Zeo (*SKN7/SKN7*::*hph*) with the plasmid pΔ*SKN7*:: *hph* . Pigmentation like wild strain	This work
Δ*rox1*$^{-/-}$	Homozygous mutant gene *ROX1*_Zeo_hyg (*ROX1*::*ble*/*ROX1*:: *hph*), obtained by transformation of the mutant strain heterozygous gene *ROX1* Zeo (*ROX1/ROX1*::*hph*) with the plasmid pΔ*ROX1*:: *hph* . Redder pigmentation than wild strain	This work
Δ*yap6*$^{-/-}$	Homozygous mutant gene *YAP6*_Zeo_Zeo (*YAP6g*:*ble*/*Yap6*:: *ble*), obtained by recombination of the mutant strain heterozygous gene *Yap6* Zeo (*Yap6/Yap6*::*ble*). Yellower pigmentation than wild strain	This work

ATCC American Type Culture Collection

B. Deletion modules

Name	Deleted gen	Integration Up pb	Integration Down pb	Deletion size pb
pBS_del-*SKN7*	*SKN7*	634	605	4319
pBS_del-*ROX1*	*ROX1*	683	648	1425
pBS_del-*YAP6*	*YAP6*	820	682	2209

C. Plasmids constructed and employed

Plasmids	Description	Reference or source
pBluescript SK-	pBluescript SK-ColE1 ori; AmpR; cloning vector with blue-white selection. Stratagene (pBS)	Stratagene
pIR-zeo	pBS contained at the EcoRV site a cassette of 1,2 kb bearing the *Streptoalloteichus hindustanus* Zeocin resistance *Sh ble* gene under the EF-1 α promoter and GPD transcription terminator of *X. dendrorhous*	[60]
pMN-*hph*	pBS contained at the EcorV site a cassette of 1,8 kb bearing the *E. coli*-hygromicin B resistance (*hph*) gene under EF-1 a promotor and the GPD transcription terminator of *X. dendrorhous.*	[2]
p∆SKN7-Xd::*ble*	pBS contained at the EcoRV, 634 pb upstream, 605 pb downstream of the *SKN7* gene and the Zeocin resistance cassette between them.	This work
p∆SKN7-Xd::*hph*	pBS contained at the EcoRV, 634 pb upstream, 605 pb downstream of the *SKN7* gene and the Hygromicin B resistance cassette between them.	This work
p∆ROX1-Xd::*ble*	pBS contained at the EcoRV, 683 pb upstream, 648 pb downstream of the *ROX1* gene and the Zeocin resistance cassette between them.	This work
p∆ROX1-Xd::*hph*	pBS contained at the EcoRV, 683 pb upstream, 648 pb downstream of the *ROX1* gene and the Hygromicin B resistance cassette between them.	This work
p∆*YAP6-Xd::ble*	pBS contained at the EcoRV, 820 pb upstream, 682 pb downstream of the *YAP6* gene and the Zeocin resistance cassette between them.	This work
p∆YAP6-Xd::hph	pBS contained at the EcoRV, 820 pb upstream, 682 pb downstream of the *YAP6* gene and the Hygromicin B resistance cassette between them.	This work

D. Primers used for module construction

del_YAP6-Fw	ACC AAG TGG ATA AAC TCT GCC
del_YAP6-HpaI-Rv	GAT CCT AAG CAT GTT AAC CGG ATG GTG TCT TGC CTG CA
del_YAP6-HpaI-Fw	ACA CCA TCC GGT TAA CAT GCT TAG GAT CGA AAC GTA G
del_YAP6-Rv	AGG TAG AGG AGT TTC CTG AGC
pre-del_YAP6-Fw	TTC CGT CTG TGT GTG TGT GAG
post-del_YAP6-Rv	TCG AGT GTA CGT TTA CCA CGT C
CTR_YAP6-Fw	ACT TGA ACA GAC CAG TCG ATG
CTR_YAP6-Rv	TTC CGA AAT GGT CGA GCT CTC
del_SKN7-Fw	TCA TCA GTG GCA GAC AGC AAC
del_SKN7-HpaI-Rv	ACC CCT CGA GGA TCG TTA ACT GAC CGG AGA GCA CTG G
del_SKN7-HpaI-Fw	TGC TCT CCG GTC AGT TAA CGA TCC TCG AGG GGT TTC C
del_SKN7-Rv	AGG TGA GAA AGG AGA GAA AGC
pre-del_SKN7-Fw	AAT CCA GGT GTC CAG GTC ATG
post-del_SKN7-Rv	AAG ATG GAT CAG AGA GTA CCC
CTR_SKN7-Fw	TCT GTG CAC TCC AAG CTC AAG
CTR_SKN7-Rv	AGG GTT GTG TTT GTA GAA GGG
del_ROX1-Fw	TTC TCT AGT GCC GTC TCA AAC
del_ROX1-HpaI-Rv	AGG ACG GGC AAG TTA ACC GAG CAA GGG CAA GCG TAG TG
del_ROX1-HpaI-Fw	TGC CCT TGC TCG GTT AAC TTG CCC GTC CTC GCC TAT TC
del_ROX1-Rv	AAT TGT TGG TTC GCG TCA GAC
pre-del_ROX1-Fw	TGG CTT CTT CCC TTT CGG TTC
post-del_ROX1-Rv	TCC ATC TCC AAC TTC GAG AAG
CTR_ROX1-Fw	TTC CGA TCT GCA CCA TCA TTC
CTR_ROX1-Rv	TGG GTG AGA AAC GAA AGC AGA G
Hyg-seq-Rv2	CAT ATC CAC GCC CTC CTA CAT
Hyg-seq-Fw2	ATG GCT GTG TAG AAG TAC TCG
p.TEF.Fw	CGGCTCATCAGCCGACAGTTCA
gpd-Rv	ACGGTTCTCTCCAAACCCTC
Zeo-F	ACGACGTGACCCTGTTCATCA
Zeo-R	TGATGAACAGGGTCACGTCGT
